# Myopia and Depression among Middle School Students in China—Is There a Mediating Role for Wearing Eyeglasses?

**DOI:** 10.3390/ijerph192013031

**Published:** 2022-10-11

**Authors:** Juerong Huang, Hongjing Dang, Yan Cai, Juan Liu, Qihui Chen

**Affiliations:** 1 College of Economics and Management, China Agricultural University, Beijing 100083, China; 2 Beijing Food Safety Policy and Strategy Research Base, China Agricultural University, Beijing 100083, China

**Keywords:** myopia, mental health, depression, eyeglasses, education, China

## Abstract

Compared with non-myopic students, myopic students face more barriers to learning (e.g., inability to see the blackboard clearly) and socializing (e.g., being victims of teasing, social exclusion and violence), which may lead to increased stress, anxiety and frustration. The high prevalence of myopia and depression among school-age children naturally raises a question of great policy relevance: are myopic students more vulnerable to mental health problems such as depression? This paper sheds some light on this question by analyzing data from the China Education Panel Survey, a large-scale survey of China’s middle school students. Our analysis first quantifies the association between myopia and sample students’ depression status (measured by the widely adopted CES-D scale) adjusted for potential confounding factors. We then explore whether the myopia–depression relationship is mediated by wearing eyeglasses, a cost-effective means of vision correction. Based on data on 19,299 middle school students, our analysis reveals that myopic students scored 0.12 standard deviations higher on the CES-D scale than their non-myopic counterparts. The adverse effect of myopia is more severe for relatively disadvantaged students: older students (who are more likely to suffer from both myopia and depression), lower-performing students and students from poorer families. Further medication analysis shows that wearing eyeglasses suppresses the myopia–depression relationship but cannot completely offset the adverse effect of myopia.

## 1. Introduction

The high prevalence of myopia among school-age children worldwide has attracted concerns about its adverse consequences from scholars, medical professionals and policy-makers [1,2,3]. Blurry vision reduces myopic students’ learning efficiency, which may also cause them to lose interest in learning, thereby undermining their school performance. As found in recent studies, myopic students are more likely to skip classes, score lower on exams and be subject to study suspension than their non-myopic counterparts [4]. Worse still, compared with non-myopic students, myopic students face more socializing barriers (e.g., being victims of teasing, social isolation and violence), which, together with poor school performance, may lead to increased stress, anxiety and frustration among the latter [4,5,6,7,8]. Yet, compared with educational outcomes [9,10,11], myopic students’ mental health problems have received much less attention.

The present study attempts to shed some light on the myopia–mental health nexus, in particular, the association between myopia and depression among middle school students in China, adjusted for potential confounding factors. Depression, a typical mental disorder, is becoming increasingly prevalent among school teens globally [12,13,14,15]. China is no exception. In China, where more than 150 million teens reside, the prevalence of depression among adolescents aged 10–19 was 24.6% in 2020 [16], very close to the global average of 25.2% estimated in a recent meta-analysis study [17]. Students with depressive symptoms were less engaged in class and more likely to suffer from other problems such as anxiety and substance abuse than those without [13,18,19,20,21]. Many health conditions, such as psychosocial stress, acute injury and chronic illness, have been identified as risk factors for adolescent depression [12,22]. It remains unclear, however, whether myopia, a commonly observed physical health condition, also acts as a risk factor for adolescent depression.

Given the rapid increases in the prevalence of both myopia and depression among Chinese teens, middle school students in China serve as an important population for studying the myopia–depression relationship. In particular, the prevalence of myopia among middle school students in 2020 (71%) was nearly twice that among primary school students (36%) in that year [16]. The prevalence of depression among middle school students was 30%, three times higher than that of primary school students [23]. Besides quantifying the myopia–depression relationship, this study pays particular attention to the role wearing eyeglasses plays in mediating this relationship. Wearing eyeglasses is proven to be an effective vision-correction method and has been shown to help improve children’s education and health outcomes [1,4,5,9,10,11,24,25]. As such, wearing eyeglasses may offset the adverse effects of myopia to some extent. On the other hand, however, wearing eyeglasses may raise concerns about one’s physical appearance and inconvenience in sports or daily activities [3], imposing more mental stress on myopic students. Therefore, whether wearing eyeglasses plays a role in the myopia–depression nexus is ultimately an empirical question. Unfortunately, the scant literature on the mediating role of wearing eyeglasses offers little insight into this question. Aiming to fill this gap, we analyze data on 19,299 middle school students from the China Education Panel Survey (CEPS), a large-scale survey of middle school (Grades 7–9) students in China. Our analysis reveals that myopic students scored 0.12 standard deviations higher on the CES-D (Center for Epidemiologic Studies Depression) scale than their non-myopic counterparts. The adverse effect of myopia is more severe for relatively disadvantaged students: older students (who are more likely to suffer from myopia and depression), lower-performing students and students from poorer families. Further medication analysis suggests that wearing eyeglasses suppresses the myopia–depression relationship but cannot completely offset the adverse effect of myopia. 

## 2. Literature Review

Existing studies on the myopia–depression relationship have focused mostly on adults and usually found that myopia is associated with higher degrees of depression among adults [26,27,28,29,30]. The rising prevalence of depression at younger ages, especially among adolescents [12,16,17], has triggered concerns about myopic children’s depression status. However, recent studies on the myopia–depression relationship in children have failed to reach a consensus. Whereas some studies reported that myopic children scored higher on depression scales [7,31,32,33,34,35], others found little effect of myopia on depression among children [36,37,38].

There are three potential explanations for the inconclusive findings on the myopia–depression relationship in children. The first is the relatively small sizes of samples used in most previous studies. Existing studies mainly relied on data from hospital records [31,32,33,36,37]. The limited scale of such data may have prevented these studies from detecting meaningful associations between children’s myopia and depression status. In fact, most existing studies with larger sample sizes (around 1000) found a significant correlation between myopia and the likelihood of exhibiting depressive symptoms among children [7,30,35,38]; in contrast, studies with a smaller sample size (<300) usually found little effect of myopia [36,37]. 

Secondly, previous findings may be tainted with estimation biases. Many existing studies failed to account for confounding factors that may bias the myopia–depression relationship, such as students’ academic performance and family environment (e.g., parental education, migration status and family wealth), which may affect both myopia and depression status. The lack of control for these potential confounders may have created room for biases (e.g., omitted-variable bias) in existing estimates [31,32,33,36,38]. 

Finally, the myopia–depression relationship might be mediated by vision-correction methods, such as wearing eyeglasses, as they ease the pressure from blurry vision and the negative feelings associated with vision impairment. If so, the actual myopia–depression relationship may depend on the proportion of children who adopted vision-correction measures in the study sample. Recent studies have shown that wearing eyeglasses not only enhances myopic students’ academic performance [1,4,10,11,24] but also leads to improvements in mental health [5] and behavioral changes [9]. Given a non-trivial proportion of myopic students in China who are reluctant to wear eyeglasses [3,10] (—nearly 30% in our data), the potential mediating effect of wearing eyeglasses is certainly worth investigating. Unfortunately, the mediating role of wearing eyeglasses has largely been overlooked in existing myopia–depression studies. 

The present study attempts to fill this gap by quantifying the association between myopia and depression among Chinese middle school students (adjusted for potential confounders) while exploring the potential role of wearing eyeglasses as a mediator in this relationship. To clearly depict the myopia–eyeglass–depression nexus, we use data from a large-scale survey (involving nearly 20,000 students) and control for a large number of factors (e.g., academic performance and family background) whose influence might have been largely overlooked in previous studies.

## 3. Materials and Methods

### 3.1. Data and Sample

Our analysis draws on publicly-available data from the China Education Panel Survey (CEPS), a large-scale survey of Grade–7 and Grade–9 students in China. The project was designed, implemented and managed by Renmin University of China (http://ceps.ruc.edu.cn/English/Home.htm, accessed on 15 October 2020). In the baseline year (2013–2014 school year), the CEPS adopted a multi-stage probability-proportional-to-size (PPS) sampling strategy to select sample students. First, 28 counties/districts in China were selected, using the average education level and proportion of migrants as stratifying variables. Next, four schools from each chosen county/district were randomly selected using enrollment size and school type (i.e., public, private and migrant children’s schools) as stratifying variables. Finally, two Grade−7 and two Grade−9 classes were randomly chosen in each selected school. All 19,487 students enrolled in the 438 selected classes were interviewed. Four rounds of follow-up surveys were conducted from 2014 to 2018, one in each academic year, but currently only two rounds of data (2013–2014 and 2014–2015) are publicly available. Our analysis focuses on the sample from the baseline survey as it has the largest size possible—only baseline 7th graders were followed in the follow-up surveys. Due to missing information, the final sample has 19,299 observations.

### 3.2. Variables

The CEPS administered separate questionnaires to sampled students, their parents and teachers, collecting information on students’ personal (e.g., age, gender and academic performance), family (e.g., parental education, migration status and family wealth) and school characteristics (e.g., teacher qualifications and conditions of school facilities). 

#### 3.2.1. Outcome Variables

The outcome variable of primary interest, students’ depression status (*D*), is measured using a shortened version of the CES-D (Center for Epidemiologic Studies Depression) scale. The CES-D scale, initially developed by Radloff (1991) [39], is one of the most frequently used instruments to measure adolescents’ and young adults’ depression status [40,41]. The CEPS version consists of five questions on students’ negative feelings during the past week: how frequently did they feel “blue/upset”/“depressed”/“unhappy”/“not enjoying life”/“sad”? Each question has five possible answers for a student to choose from: “never” (=0), “seldom” (=1), “sometimes” (=2), “often” (=3) and “always” (=4). The final scores were obtained by summing the scores of all five questions. The resulting scores range from 0 to 20; a higher score reflects a more severe depression status.

To assess the robustness of our findings, we also consider a set of related outcomes: self-confidence in one’s future, motivation for schooling and perceptions of school life. Specifically, “self-confidence” (*D_S_*) is measured by answers to the question: “Do you feel confident in your future?” Possible answers include: “not confident at all” (=0), “not so confident” (=1), “somewhat confident” (=2) and “very confident” (=3). “Motivation for schooling” (*D_M_*) is measured by summing a student’s responses to three statements: “I would try my best to: (i) attend school even if not feeling well or having other reasons to stay home; (ii) finish my homework I dislike; (iii) finish my homework even if it would take me a long time.” Possible answers include: “strongly disagree” (=0), “somewhat disagree” (=1), “somewhat agree” (=2) and “strongly agree” (=3). Finally, variables related to students’ “perceptions of school life” (*D_P_*) are measured by their responses to the following six statements: “Most of my classmates are nice to me”; “I think I am easy to get along with”; “My class is in a good atmosphere”; “I often take part in school/class activities”; “I feel close to people in this school”; “I feel bored at school”. The responses range from 0 (“strongly disagree”) to 3 (“strongly agree”) for each statement. These responses are used as separate outcome variables in some analyses below.

#### 3.2.2. Explanatory Variable of Primary Interest

Myopic status (*M*), the explanatory variable of primary interest, is recorded by students’ self-reported status—a binary indicator of whether they were reportedly myopic at the time of the survey (=1 if yes; =0 if no). The prevalence of myopia is 60.4% among all sampled students: 54.8% among 7th graders and 66.8% among 9th graders.

#### 3.2.3. Mediating Variable

Wearing eyeglasses (*G*), the mediating variable in our study, is a binary indicator created based on students’ self-reported myopia status—the CEPS specifically asked sample students about their “myopia” status rather than “vision impairment”—and their reported refractive errors. All sampled students who were reportedly myopic either further reported specific refractive errors in their eyes (in diopters) from their eyeglasses prescription or responded that they were “not sure” about their refractive errors. Given these responses, a student is considered “wearing eyeglasses” (*G* = 1) if he/she reported specific refractive errors and “not wearing eyeglasses” (*G* = 0) if he/she was “not sure” about the negative refractive errors. Among all 11,665 myopic students in our sample, only 71.2% reported wearing eyeglasses: 69.9% among 7th graders and 72.3% among 9th graders.

#### 3.2.4. Other Covariates

To address potential confounding (at least partially), we follow existing studies [12,42] and control for students’ personal and family characteristics that have been found to be correlated with myopia and may influence depression in our analysis. Personal-level covariates include age, gender, sibship size, grade and test scores of core subjects (Chinese, Math and English) in China’s middle school curricula that are involved in all important exams prior to tertiary education, such as high school and college entrance exams, in China. Family-level covariates include sibship size, parents’ years of education, migrant status, residential area and household income.

### 3.3. Method

#### 3.3.1. Conceptual Framework

**Figure 1:**
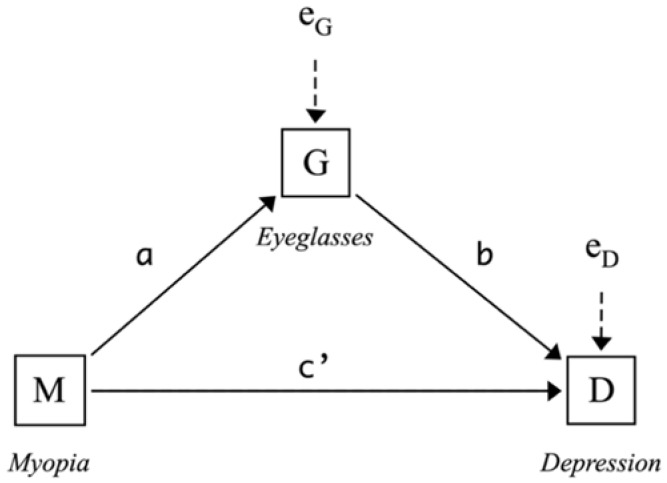
Conceptual framework.

In our context, whether to wear eyeglasses becomes a question only when one is myopic. Thus, wearing eyeglasses, if indeed playing a role, will be located somewhere between myopia and depression in the myopia–depression relationship. Myopia (*M*) may affect one’s depression status (*D*) through two pathways (Figure 1): the direct path from *M* to *D* without passing through wearing eyeglasses (*G*) and the indirect path from *M* to *D* through *G*, the mediator of interest.

Correspondingly, myopia potentially exerts two effects, one direct and one indirect, on depression. As illustrated in Figure 1, the direct effect, represented by $c^{\prime }$, can be defined formally as

(1) $$c^{\prime }=\left[\bar{D}|M=1;G=g\right]-\left[\bar{D}|M=0,G=g\right],$$

where $\bar{D}$ is the mean of *D* for students who fit the descriptions specified behind the “|” symbol; g is any given value of G (implying that G is being held constant). In words, $c^{\prime }$ is the mean difference in *D* between myopic (*M* = 1) and non-myopic (*M* = 0) students holding *G* constant.

Before defining the indirect effect, it is helpful to discuss first the meanings of the parameters a and b: a represents the mean difference in *G* between myopic and non-myopic students.

(2) $$a=\left[\bar{G}|M=1\right]-\left[\bar{G}|M=0\right];$$

b represents the mean difference in *D* between students with and without wearing eyeglasses (*G*) conditional on M:

(3) $$b=\left[\bar{D}|G=1;M=m\right]-\left[\bar{D}|G=0;M=m\right],$$

where m is any value of M (i.e., M is held constant). Given these definitions, the indirect effect of *M* on *D* through *G* is simply the product of a and b: (a×b) [43].

The total effect, c, quantifies how much *D* differs between the two groups defined by different values of *M*, on average, i.e., $c=\left[\bar{D}|M=1\right]-[\bar{D}|M=0]$. Since the effect of *M* on *D* is partitioned into direct and indirect components, the total effect can be written as the sum of direct and indirect effects:

(4) $$c=c^{\prime }+a\times b$$

#### 3.3.2. Mediation Model

To estimate the effects of myopia discussed above, we transform the above conceptual framework (Figure 1) into the following linear regression models:

(5) $$G=i_{G}+aM+Xa_{x}+e_{G},$$

(6) $$D=i_{D}+c^{\prime }M+bG+Xc_{x}^{\prime }+e_{D},$$

where *G*, *D* and *M* have been defined above; X represents a set of observed covariates (discussed in Section 3.2.4); $i_{G}$ and $i_{D}$ are constant terms; $e_{G}$ and $e_{D}$ capture the influence of unobserved factors and random disturbances in the models for G and D, respectively.

As noted above, the parameters of primary interest are $c^{\prime }$—the direct effect of myopia on depression, $\left(a\times b\right)$—the indirect effect of myopia through wearing eyeglasses and $c\;\left(=c^{\prime }+a\times b\right)$—the total effect of myopia. Note that the total effect of myopia on depression, *c*, can be directly obtained by regressing one’s depression scale (*D*) on myopia status (*M*) and the set of covariates (***X***), leaving out the mediating variable *G*:

(7) $$D=i_{D*}+cM+Xc_{x}+e_{D*},$$

To see this, simply substitute Equation (5) into (6), expressing *D* as a function of only *M* (and ***X***) and collecting terms to yield:

(8) $$D=(i_{D}+b\times i_{G})+\left(c^{\prime }+a\times b\right)M+X\left(c_{x}^{\prime }+b\times a_{x}\right)+\left(e_{D}+b\times e_{G}\right).$$

Note that the role of wearing eyeglasses in the myopia–depression relationship depends on whether the indirect effect $\left(a\times b\right)$ is present and, if so, its sign compared with that of the direct effect $c^{\prime }$. If a×b=0, the total effect of myopia reduces to its direct effect: $c=c^{\prime }$. If a×b≠0, the mediating effect of wearing eyeglasses is at work, i.e., serving as a channel through which myopia affects depression. If (a×b) and $c^{\prime }$ are of the same sign, wearing eyeglasses has a partial mediating effect in the myopia–depression relationship; if (a×b) and $c^{\prime }$ have opposite signs, wearing eyeglasses has a suppressing effect on this relationship [43].

If Equations (5) and (6) are correctly specified, these three parameters (or at least their components) can all be estimated by OLS techniques. All effects except for the indirect effect (a×b) can be tested in a straightforward manner. The indirect effect (a×b) is commonly tested using bootstrapping techniques [43,44]. By randomly sampling observations from the original sample with replacements (500 replications in this study), the standard errors of ($\hat{a\times b}$) and a 95% bootstrap confidence interval (CI) around the estimate ($\hat{a\times b}$) are obtained for statistical inference. 

All analyses in this study were performed using Stata SE 15 (StataCorp LCC, College Station, TX, USA).

## 4. Results

### 4.1. Descriptive Analysis

Table 1 presents summary statistics for the full sample of students (column 1) and separately for non-myopic (column 2) and myopic students (column 3). Among all sampled students, 48.6% are female. An average student was 14.1 years old, with 0.75 siblings. Slightly more than half (54.8%) of all students were rural residents. Sampled students’ fathers and mothers, respectively, completed 10.2 and 9.4 years of schooling on average; 19.8% of fathers and 13.9% of mothers were migrant workers at the time of the survey. Nearly three-quarters (73.1%) of the students (and their parents) reported that their household income was at the “average” level.

**Figure 2:**
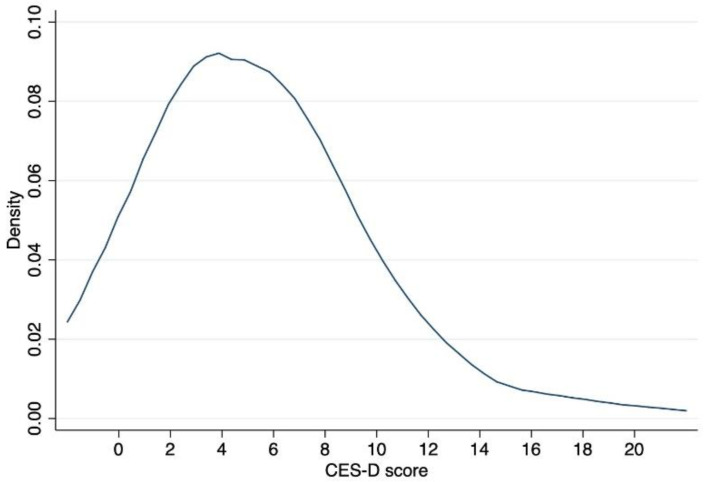
Distribution of depression (CES-D) scores.

Turning to the outcome of primary interest, Figure 2 plots the distribution of sample students’ depression (CES-D) scores. While the average score of 5.42 seems relatively low, the standard deviation (SD) of 4.11 suggests that there is much variation in these scores to be explained by other factors. For ease of interpretation, we use the standardized CES-D scores with zero mean and unit SD within schools in the analysis below. 

Table 2 reports the standardized CES-D scores and other outcome variables for all sampled students (column 1) and separately by their myopic status (columns 2–3). Columns 2–4 suggest that myopic students scored higher on the CES-D scale (i.e., exhibiting more depressive symptoms) than non-myopic students (*p* < 0.01). Yet, somewhat counterintuitively, myopic students also reported a better perception of school life than non-myopic students (*p* < 0.01). One reason for this puzzling finding is that the above bivariate relationships may be tainted by confounding factors, such as academic performance and family background, that simultaneously influence one’s myopic and mental health status. In fact, columns 2–4 reveal many statistically significant differences in personal and family characteristics between myopic and non-myopic students, suggesting the need to control for potential confounders when depicting the myopia–depression relationship.

### 4.2. The Total Effect of Myopia on Depression

Table 3 reports the main results of our meditation analysis. Columns (1) and (2) present the estimates of the total effect of myopia (i.e., captured by the coefficient c in Equation (7)) on students’ depression (CES-D) scores. Column (1) suggests that myopic students scored significantly higher on the CES-D scale than their non-myopia counterparts (by 0.135 SDs; *p* < 0.01), suggesting that the former suffer more from depression problems. The estimate remains similar ($\hat{c}$ = 0.120 SDs; *p* < 0.01) when a large set of student and family characteristics (Table 1) are included in the model (column 2), greatly alleviating the concern that the estimated total effect of myopia might be confounded by other factors.

### 4.3. Direct and Indirect Effects of Myopia on Depression

Columns (3) and (4) of Table 3 present the estimates of the direct effects of myopia (i.e., $c^{\prime }$ in Equation (6)) and wearing eyeglasses (i.e., b in Equation (6)) on students’ depression scores. As shown in column (3), the estimate of $c^{\prime }$ suggests that the difference between myopic and non-myopic students’ CES-D scores becomes even larger ($\hat{c^{\prime }}$= 0.181 SDs; *p* < 0.01) after adjusting for the effect of wearing eyeglasses (*G*), suggesting wearing eyeglasses offsets part of the adverse effect of myopia on depression. Indeed, the estimate of b (the coefficient of “wearing eyeglasses”) reveals a negative association between wearing eyeglasses and students’ CES-D scores (column 3: $\hat{b}$ = −0.067 SDs; *p* < 0.01), i.e., myopic students wearing eyeglasses are less depressed than those not wearing eyeglasses. These estimates remain robust after controlling for students’ personal and family characteristics (column 4: $\hat{c^{\prime }}$= 0.155 SDs, *p* < 0.01; $\hat{b}$ = −0.053 SDs, *p* < 0.05).

Columns (5)–(6) report the estimated indirect effect of myopia (i.e., a×b) on depression through wearing eyeglasses. Column (5) shows that myopic students wearing eyeglasses scored lower on the CES-D scale ($\hat{a\times b}$ = −0.046 SDs; *p* < 0.05), with a 95% bootstrap confidence interval entirely below zero (95% CI = (−0.085, −0.008)). The estimate also remains similar when controlling for students’ personal and family characteristics (column 6: $\hat{a\times b}$ = −0.036 SDs; *p* < 0.1). The opposite signs of the direct effect (columns 3–4) and the indirect effect of myopia (columns 5–6) suggest that wearing eyeglasses “suppresses” the myopia–depression relationship. Nevertheless, its suppressing effect is not large enough to completely offset the adverse effect of myopia—the total effect still suggests that being myopic exacerbates students’ depression status (columns 1–2).

**Figure 3:**
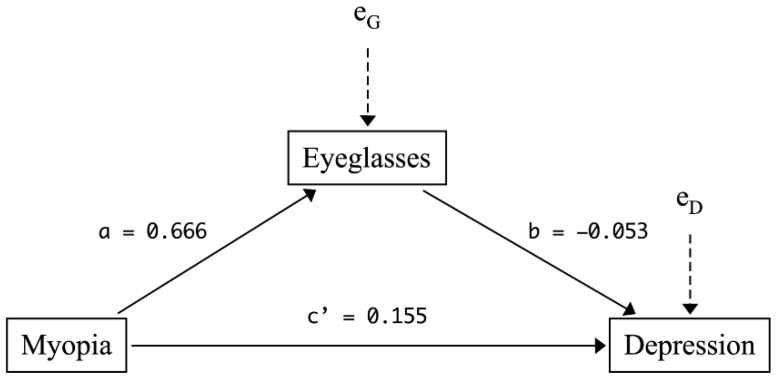
Mediation model for the myopia–depression nexus, adjusted for student and family characteristics.

Figure 3 summarizes all three effects (total, direct and indirect effects) of myopia on students’ depression scores and the mediating effect of wearing eyeglasses discussed above, adjusted for the set of student and family characteristics reported in Table 1.

### 4.4. Robustness Checks

Before discussing our findings further, it is helpful to rule out potential threats to our main findings and thus strengthen our findings. 

#### 4.4.1. Additional Control Variables

Even though the estimates of the three effects discussed above (Table 3) are robust in relation to the inclusion of the set of personal and family characteristics, one might still be concerned about the potential existence of other confounding factors that may drive the myopia–depression relationship. Since being myopic is likely to affect students’ daily activities [9], this subsection further examines the potential influence of students’ time spent on various activities, including sleeping, outdoor activities/sports, watching television and surfing the Internet/playing video games. 

Table 4 reports the estimates of the total effect of myopia (i.e., *c* in Equation (7)) obtained after controlling for these additional (activity-related) covariates in the model, besides the set of personal- and family-level covariates used above. Note that while these newly-added covariates are themselves significantly correlated with students’ depression scores (at least at the *p* < 0.1 level), the new estimates of the total effect of myopia remain highly comparable to our original estimate (Table 3, column 2). The similarity in estimates lends further support to our main findings discussed above.

#### 4.4.2. Related Outcomes

Another concern is that the outcome variable examined above, students’ scores on the simplified version of the CES-D scale, are too crude to capture the real myopia–eyeglasses–depression nexus. This subsection thus examines a set of other outcomes that are presumably correlated with students’ depression status, including confidence in one’s future, motivation for schooling and perceptions of school life (discussed in Section 3.2.1), to see whether the patterns we discovered for CES-D scores apply to these additional outcomes as well. Table 5 reports the main results.

Three notable findings emerge. First, regarding the total effect of myopia, panel A of Table 5 suggests that myopia exerts an adverse effect on all related outcomes examined (except for the perceptions of “often taking part in school/class activities” and “feeling bored at school”, for which the effects are statistically insignificant). More specifically, compared with their non-myopic counterparts, myopic students feel less confident in their future (column 1) and are less motivated to attend school and complete school assignments (column 2). Likewise, they perceive school life more negatively: they are more likely to struggle socializing with peers (columns 3–5) and feel more isolated at school (column 7).

Second, concerning the direct effect of myopia, as with columns (3)–(4) of Table 3, panel B of Table 5 reveals an even larger adverse effect of myopia on all related outcomes after adjusting for the influence of wearing eyeglasses. Note that myopic students not wearing eyeglasses are also less likely to participate in school/class activities (column 6) and are more likely to feel bored at school (column 8), but these effects are, to some extent, offset by wearing eyeglasses (Panel A).

Third, reporting the indirect effect of myopia on depression through wearing eyeglasses, panel C of Table 5 reveals that myopic students wearing eyeglasses fare better than those not wearing eyeglasses on all outcomes examined. In particular, myopic students wearing eyeglasses feel more confident in their future (column 1), are more likely to feel that classmates were nice to them (column 3) and more likely to participate in school/class activities (column 6). The differences are statistically significant (*p* < 0.05). These findings further support the role wearing eyeglasses plays as a mediator in the myopia–depression nexus—this mediator suppresses some of the adverse effects of myopia on the outcomes examined in this subsection.

#### 4.4.3. Heterogeneity in the Myopia–Depression Relationship

Note that the effects of myopia discussed above only speak to the “average” student, which might have masked important heterogeneity in the myopia–depression relationship. To further examine how the effects of myopia may differ across student subgroups, Table 6, Table 7 and Table 8 report estimates of the effects of myopia for subsamples of students defined by their demographic characteristics, academic performance and family characteristics. Several informative patterns emerge. The direct and total (adverse) effects of myopia are stronger for relatively disadvantaged students: older students—who are more likely to suffer from myopia and depression (Table 6, columns 3–6), lower-performing students (Table 7, columns 1–6) and students from poorer families (Table 7, columns 7–9); for these subgroups, the indirect effects of myopia (through wearing eyeglasses) are rather limited (and mostly statistically insignificant). No significant differences in the effects of myopia were detected across gender groups (Table 6, columns 1–2), sibship sizes (Table 8, columns 1–2) and parental education levels (Table 8, columns 3–6). The finding that relatively disadvantaged students suffer more from the adverse effects of myopia naturally implies that more attention should be paid to these students.

## 5. Discussion

This study sought to answer the following questions: Are myopic students more vulnerable to depression? If so, is wearing eyeglasses a mediator in the myopia–depression nexus? If wearing eyeglasses does play a role, what role does it play? Based on data on 19,299 middle school students in China, our analysis reveals that myopic students scored higher on a widely-adopted depression scale (CES-D)—i.e., they suffered from more depressive symptoms—than non-myopic students. Moreover, the direct and total (adverse) effects of myopia are larger for relatively disadvantaged students: older students (who suffer more from both myopia and depression), lower-performing students and students from poorer families. While wearing eyeglasses suppresses the myopia–depression relationship, it lacks the power to completely offset the adverse direct effect of myopia. Given these findings, a number of related points merit further discussions.

Depression in adolescents is becoming increasingly more prevalent but often overlooked by parents. Because adolescence is a typical period characterized by emotional instability, parents may underestimate the likelihood of depression in their children and fail to link depression to related physical symptoms, such as eating disorders and refusal to attend school [40,45]. Since depression in adolescence predicts a range of mental disorders, physical health problems and even suicidal behaviors in adulthood [12,13], it is worth emphasizing the need for more attention to physical symptoms that may potentially be linked to adolescent depression. Meanwhile, to the extent that adolescents are the future of society, the physical symptoms–depression relationship also helps predict the mental state of future society. (We thank an anonymous reviewer for suggesting this point).

Among risk factors of adolescent depression, myopia has been underexplored. The findings of this study underscore the importance of more attention being paid to depressive symptoms among myopic adolescents. We found that, in addition to depression, myopic adolescents feel less confident in their future, are less motivated for schooling and perceive school life more negatively than their non-myopic counterparts, adding new evidence of the adverse effects of myopia on mental health among children (see more discussion on this below). Together with these related findings, our finding of a robust association between myopia and depression also highlights the importance of formulating policies and measures to help reduce myopia prevalence among school-age children. The studies by Li et al. (2022) [7] and Huang et al. (2020) [46] provide recent examples.

The results of our mediation analyses also highlight the importance of understanding the role wearing eyeglasses plays as a mediator in the myopia–depression relationship. While many previous studies have found null or weak effects of myopia on adolescent depression [36,37,38], these findings might be due to the existence of wearing eyeglasses as a suppressing mediator in the myopia–depression relationship. The suppressing effect of wearing eyeglasses also suggests the need to further study why some myopic students are reluctant to wear eyeglasses and what can be done to urge these students to wear them. It is worth pointing out that—although our study does not provide evidence to support this point—it is also possible that wearing eyeglasses may cause a high probability of depression, as wearing and taking care of one’s eyeglasses may be a nuisance in daily life for some students. (We thank an anonymous reviewer for raising this point). In any case, the role of wearing eyeglasses in the myopia–depression relationship merits further exploration.

Before drawing conclusions, a note on the limitations of this study is in order. First, despite our efforts to address potential confounding factors (in particular, by including a large number of control variables in the models presented in Table 3 and Table 4), the estimated relationships reported in this study may still not be causal. For example, some potential confounders (e.g., genetic or environmental factors that affect both myopia and depression) are difficult to measure; even the large set of control variables included in our models may not be able to completely net out the influence of confounders. Some covariates included in our models (e.g., students’ academic performance) might be outcomes of depression, which may also be sensitive to potential causal factors of myopia (e.g., family income and parental education) included in the model. They might also have a complex or bidirectional association with depression—for example, academic performance may be a “collider” in the myopia–depression relationship. (We thank an anonymous reviewer for raising these points). These issues might render such covariates “bad controls” [47], the inclusion of which might bias the myopia–depression relationship. 

An ideal and comprehensive approach would be to develop the full structural model as directed acyclic graphs [48] and collect all relevant factors needed to tease out the causal effects of interest. Alternatively, partial causal models that employ quasi-experimental designs may be used to establish causality. Unfortunately, the CEPS data available to us lack sufficient information for us to pursue further in these directions. On a more positive note, we estimated models with and without these covariates. The similarity in the results with and without these covariates greatly alleviated the concerns about them being “bad controls”. Even if the relationships are not causal, the significant associations we estimated in the study merit attention from academics and policy-makers. For example, if some confounding factors drive our observed myopia–depression relationship, such factors must be correlated with both myopia and depression. At a minimum, this implication points out the direction for future studies to locate such factors. Moreover, we believe that our study has provided a systematic framework to understand the myopia–eyeglass–depression nexus and can attract studies that focus on identifying causal effects (perhaps with better data and/or more sophisticated methods). Since wearing eyeglasses affects not only child education but also health, future explorations of the mediating effects of wearing eyeglasses on the myopia–education-health nexus are expected to be fruitful. 

Second, the key explanatory and mediating variables, i.e., myopia and wearing eyeglasses, are measured by sampled students’ self-reported answers, which are too crude to rule out some important measurement issues. For example, under-correction of refractive errors is possible for some students who self-reported wearing eyeglasses, which could limit the mediating effect of wearing eyeglasses. (We thank an anonymous reviewer for pointing this out). Given our finding that wearing eyeglasses lowers students’ depression (CES-D) scores, our estimation provided a conservative estimate of the eyeglasses-wearing effect if there were some students whose eyeglasses failed to correct their vision perfectly. In other words, the beneficial impact of wearing eyeglasses is presumably greater if all students obtained clear vision through eyeglasses. Another measurement issue is that myopic students who reported “not sure” about the refractive errors from their eyeglass prescriptions were defined as “not wearing eyeglasses”, which might have misclassified some eyeglasses-wearing students as not wearing eyeglasses. Yet this problem may not be as serious as it seems. To the extent that wearing eyeglasses helps reduce depression (as we found), this misclassification problem is likely to underestimate the medicating role of wearing eyeglasses in suppressing the myopia–depression relationship, which, in effect, helps strengthen our findings.

Finally, our data lack precise information on the onset and progression of myopia, but children developing myopia at an early age might be more prone to mental illnesses. Thus, pooling children with different histories of myopia may lead to different findings than those obtained conditional on these histories. While our data lack the power to explore further along these lines, we certainly hope that future studies may benefit from collecting more detailed data or employing more specific measures to more accurately capture children’s visual acuity (e.g., through medical examinations), eyeglasses-wearing status and their relationship.

## 6. Conclusions

Using data on 19,299 middle school students in China from the China Education Panel Survey, our mediation analysis reveals that myopic students are significantly more prone to depression than non-myopic students. Even though wearing eyeglasses serves to suppress the myopia–depression relationship to some extent, it cannot completely offset the detrimental effect of myopia. Three policy implications are immediate. First, more attention should be paid to the mental health status of myopic children, who are more vulnerable to depression. Second, the need for prompt correction for myopia through wearing properly-fitted eyeglasses should be emphasized, which is expected to weaken the link between myopia and depression. Finally, measures and interventions that may help reduce myopia prevalence are needed to prevent the adverse effect of myopia from occurring.

## Figures and Tables

**Table 1:** Summary statistics of student and family characteristics, by myopic status.

	(1)	(2)	(3)	(4)
	All Students	Non-Myopic	Myopic	Difference = (3) − (2)
A. Student characteristics				
Male	0.514	0.574	0.475	−0.100 ***
	[0.500]	[0.490]	[0.499]	(0.007)
Age (years)	14.108	14.011	14.172	0.162 ***
	[1.252]	[1.273]	[1.234]	(0.018)
Grade 9	0.474	0.398	0.524	0.126 ***
	[0.499]	[0.490]	[0.499]	(0.007)
Standardized Chinese score	0.006	−0.104	0.078	0.183 ***
	[0.981]	[1.014]	[0.951]	(0.015)
Standardized math score	0.005	−0.083	0.062	0.146 ***
	[0.984]	[1.004]	[0.966]	(0.015)
Standardized English score	0.006	−0.106	0.078	0.184 ***
	[0.983]	[0.995]	[0.976]	(0.015)
B. Family characteristics				
Father’s education (years)	10.147	9.715	10.430	0.715 ***
	[3.128]	[2.948]	[3.208]	(0.046)
Mother’s education (years)	9.370	8.883	9.689	0.806 ***
	[3.499]	[3.445]	[3.497]	(0.051)
Father is a migrant worker	0.198	0.223	0.181	−0.042 ***
	[0.398]	[0.416]	[0.385]	(0.006)
Mother is a migrant worker	0.139	0.165	0.123	−0.043 ***
	[0.346]	[0.372]	[0.328]	(0.005)
Sibship size	0.748	0.864	0.672	−0.192 ***
	[0.822]	[0.848]	[0.796]	(0.012)
Rural residents	0.548	0.612	0.506	−0.106 ***
	[0.498]	[0.487]	[0.500]	(0.007)
Household income:				
Very poor	0.038	0.046	0.032	−0.014 ***
	[0.190]	[0.209]	[0.176]	(0.003)
Poor	0.171	0.203	0.150	−0.053 ***
	[0.377]	[0.402]	[0.357]	(0.005)
Average	0.731	0.695	0.755	0.060 ***
	[0.443]	[0.460]	[0.430]	(0.007)
Rich	0.057	0.051	0.060	0.009 **
	[0.231]	[0.221]	[0.238]	(0.004)
Very rich	0.003	0.004	0.003	−0.002
	[0.057]	[0.064]	[0.052]	(0.001)
*N*	19,299	7634	11,665	

Notes: The sample size reported (*N*) refers to the maximum number of observations in the respective samples; the actual sample sizes used in the analysis below are slightly smaller due to missing values in some variables. Standard deviations (errors) in brackets (parentheses). *** *p* < 0.01, ** *p* < 0.05.

**Table 2:** Summary statistics of outcome variables, by myopic status.

	(1)	(2)	(3)	(4)
	All Students	Non-Myopic	Myopic	Difference = (3) − (2)
A. Depression scale: standardized CES-D scores	−0.001	−0.077	0.049	0.126 ***
	[0.998]	[0.983]	[1.005]	(0.015)
B. Feeling confident in one self’s future (range: 0–3)	2.212	2.209	2.214	0.005
	[0.720]	[0.726]	[0.716]	(0.011)
C. Motivation for schooling (range: 0–9)	6.930	6.954	6.915	−0.040
	[2.012]	[1.980]	[2.033]	(0.030)
D. Perceptions of school life (range: 0–3 for each statement below)				
Most classmates are nice to me.	2.262	2.233	2.281	0.047 ***
	[0.807]	[0.823]	[0.796]	(0.012)
I think I am easy to get along with.	2.163	2.137	2.179	0.043 ***
	[0.842]	[0.856]	[0.833]	(0.013)
My class has a good atmosphere.	2.134	2.095	2.159	0.065 ***
	[0.877]	[0.895]	[0.864]	(0.013)
I often take part in school/class activities.	1.744	1.692	1.779	0.088 ***
	[1.017]	[1.032]	[1.006]	(0.015)
I feel close to people in this school.	1.932	1.890	1.959	0.069 ***
	[0.926]	[0.943]	[0.914]	(0.014)
I feel bored at school.	0.678	0.698	0.665	−0.034 ***
	[0.871]	[0.890]	[0.859]	(0.013)
N	19,154	7570	11,584	

Notes: The sample sizes reported in the table refer to the maximum number of observations in the respective samples; the actual sample sizes used in the analysis below are somewhat smaller due to missing values in some of the variables. Standard deviations (errors) are reported in brackets (parentheses). *** *p* < 0.01.

**Table 3:** Meditation analysis of the effect of myopia on depression.

	(1)	(2)	(3)	(4)	(5)	(6)
	Total Effect		Direct Effect		Indirect Effect	
Outcome:	CES-D Scores	CES-D Scores	CES-D Scores	CES-D Scores	CES-D Scores	CES-D Scores
Myopic (*M*)	0.135 ***	0.120 ***	0.181 ***	0.155 ***	−0.046 **	−0.036 *
	(0.017)	(0.017)	(0.021)	(0.020)	(0.020)	(0.019)
95% (bootstrap) CI	[0.102, 0.168]	[0.086, 0.153]	[0.140, 0.222]	[0.114, 0.195]	[−0.085, −0.008]	[−0.073, −0.003]
Wearing eyeglasses (*G*)			−0.067 ***	−0.053 **		
			(0.022)	(0.021)		
95% (bootstrap) CI			[−0.110, −0.025]	[−0.095, −0.010]		
Control variables	no	yes	no	yes	no	yes
School fixed effects	yes	yes	yes	yes	yes	yes
N	18,645	18,645	18,645	18,645	18,645	18,645
R^2^	0.004	0.030	0.005	0.030		

Notes: “Control variables” include all student and family characteristics presented in Table 1. Standard errors, reported in parentheses, are clustered at the school level. 95% confidence intervals (CIs) are reported in brackets; for the indirect effect estimates (columns 5–6), 95% bootstrap CIs are reported. *** *p* < 0.01, ** *p* < 0.05, * *p* < 0.1.

**Table 4:** Estimates of the total effect of myopia on students’ depression (CES-D) scores with additional control variables.

	(1)	(2)	(3)	(4)	(5)
Myopic	0.116 ***	0.119 ***	0.123 ***	0.119 ***	0.117 ***
	(0.017)	(0.017)	(0.017)	(0.017)	(0.017)
Time allocation (hours/day):					
Sleep	−0.048 ***				−0.049 ***
	(0.006)				(0.006)
Outdoor activities/sports		−0.015 *			−0.027 ***
		(0.008)			(0.008)
Watching television			0.029 ***		0.023 ***
			(0.006)		(0.006)
Internet surfing/playing video games				0.042 ***	0.038 ***
				(0.008)	(0.008)
Personal- and family-level control variables	yes	yes	yes	yes	yes
School fixed effects	yes	yes	yes	yes	yes
N	18,645	18,645	18,645	18,645	18,645
R^2^	0.036	0.030	0.032	0.034	0.043

Notes: “Personal- and family-level control variables” include all student and family characteristics presented in Table 1. Standard errors, reported in parentheses, are clustered at the school level. *** *p* < 0.01, * *p* < 0.1.

**Table 5:** Meditation analysis of the effect of myopia on other outcomes.

	(1)	(2)	(3)	(4)	(5)	(6)	(7)	(8)
Outcomes:	Feeling Confident in My Future	Motivation for Schooling	Perceptions of School Life					
			Most of My Classmates Are Nice to Me	I Think I Am Easy to Get Along With	My Class Isin a GoodAtmosphere	I Often TakePart in School/ Class Activities	I Feel Close to the People in This School	I Feel Bored at School
	A. Total Effects							
Myopic	−0.029 **	−0.091 ***	−0.035 ***	−0.034 **	−0.026 *	−0.024	−0.024 *	0.018
	(0.011)	(0.030)	(0.012)	(0.015)	(0.015)	(0.016)	(0.014)	(0.013)
95% CI	[−0.052, −0.007]	[−0.151, −0.031]	[−0.060, −0.010]	[−0.063, −0.005]	[−0.055, 0.003]	[−0.056, 0.008]	[−0.052, 0.003]	[−0.008, 0.044]
	B. Direct Effects							
Myopic	−0.060 ***	−0.133 ***	−0.067 ***	−0.062 ***	−0.043 **	−0.070 ***	−0.049 **	0.045 **
	(0.017)	(0.043	(0.016)	(0.018)	(0.019)	(0.022)	(0.021)	(0.018)
95% CI	[−0.094, −0.026]	[−0.218, −0.048]	[−0.099, −0.034]	[−0.099, −0.025]	[−0.080, −0.006]	[−0.115, −0.026]	[−0.090, −0.008]	[0.009, 0.081]
	C. Indirect Effects							
Myopic	0.031 **	0.042	0.031 **	0.027	0.015	0.044 **	0.025	−0.027
	(0.014)	(0.038)	(0.015)	(0.016)	(0.017)	(0.020)	(0.017)	(0.019)
95% bootstrap CI	[0.003, 0.057]	[−0.029, 0.121]	[0.001, 0.060]	[−0.007, 0.060]	[−0.019, 0.053]	[0.002, 0.081]	[−0.005, 0.058]	[−0.067, −0.010]
N	19,154	18,555	19,103	19,108	19,077	19,085	18,954	19,008

Notes: All regressions include all student and family characteristics presented in Table 1 and school fixed effects. Standard errors, reported in parentheses, are clustered at the school level. 95% confidence intervals (CIs) are reported in brackets; for the indirect effect estimates, 95% bootstrap CIs are reported. *** *p* < 0.01, ** *p* < 0.05, * *p* < 0.1.

**Table 6:** Subgroup analysis, by students’ demographic characteristics.

	(1)	(2)	(3)	(4)	(5)	(6)
Subsample	Female	Male	Age ≤ 14	Age > 14	Grade 7	Grade 9
	A. Total Effects					
Myopia	0.111 ***	0.127 ***	0.082 ***	0.163 ***	0.094 ***	0.153 ***
	(0.025)	(0.024)	(0.023)	(0.022)	(0.022)	(0.022)
95% CI	[0.063, 0.160]	[0.079, 0.175]	[0.037, 0.128]	[0.120, 0.206]	[0.050, 0.139]	[0.108, 0.198]
	B. Direct Effects					
Myopia	0.144 ***	0.165 ***	0.140 ***	0.183 ***	0.148 ***	0.181 ***
	(0.029)	(0.031)	(0.033)	(0.028)	(0.032)	(0.028)
95% CI	[0.086, 0.203]	[0.104, 0.225]	[0.075, 0.206]	[0.128, 0.238]	[0.085, 0.211]	[0.125, 0.237]
	C. Indirect Effects					
Myopia	−0.033	−0.038	−0.060 *	−0.020	−0.054 *	−0.029
	(0.026)	(0.030)	(0.031)	(0.026)	(0.029)	(0.027)
95% bootstrap CI	[−0.088, 0.014]	[−0.100, 0.024]	[−0.119, 0.004]	[−0.075, 0.030]	[−0.114, 0.002]	[−0.083, 0.024]
N	9105	9540	9004	9641	9737	8908

Notes: All regressions include all personal and family characteristics presented in Table 1 and school fixed effects. Standard errors, reported in parentheses, are clustered at the school level. 95% confidence intervals (CIs) are reported in brackets; for indirect effect estimates, 95% bootstrap CIs are reported. *** *p* < 0.01, * *p* < 0.1.

**Table 7:** Subgroup analysis, by students’ academic performance.

	(1)	(2)	(3)	(4)	(5)	(6)
Subsample	Standardized Chinese score ≤ median	Standardized Chinese score > median	Standardized math score ≤ median	Standardized math score > median	Standardized English score ≤ median	Standardized English score > median
	A. Total Effects					
Myopia	0.140 ***	0.096 ***	0.133 ***	0.104 ***	0.133 ***	0.103 ***
	(0.023)	(0.022)	(0.027)	(0.020)	(0.025)	(0.020)
95% CI	[0.094, 0.187]	[0.053, 0.139]	[0.080, 0.186]	[0.065, 0.144]	[0.083, 0.183]	[0.063, 0.143]
	B. Direct Effects					
Myopia	0.161 ***	0.142 ***	0.186 ***	0.116 ***	0.183 ***	0.117 ***
	(0.029)	(0.026)	(0.033)	(0.026)	(0.032)	(0.029)
95% CI	[0.103, 0.219]	[0.089, 0.194]	[0.121, 0.252]	[0.065, 0.167]	[0.120, 0.245]	[0.060, 0.174]
	C. Indirect Effects					
Myopia	−0.027	−0.040	−0.055 **	−0.011	−0.051 **	−0.014
	(0.024)	(0.026)	(0.026)	(0.026)	(0.025)	(0.029)
95% bootstrap CI	[−0.075, 0.026]	[−0.095, 0.008]	[−0.107, −0.008]	[−0.061, 0.039]	[−0.098, −0.004]	[−0.069, 0.044]
N	9189	9456	9187	9458	9207	9438

Notes: All regressions include all personal and family characteristics presented in Table 1 and school fixed effects. Standard errors, reported in parentheses, are clustered at the school level. 95% confidence intervals (CIs) are reported in brackets; for indirect effect estimates, 95% bootstrap CIs are reported. *** *p* < 0.01, ** *p* < 0.05.

**Table 8:** Subgroup analysis, by family characteristics.

	(1)	(2)	(3)	(4)	(5)	(6)	(7)	(8)	(9)
Sub-sample	No siblings	With siblings	Father’s education ≤ 9	Father’s education > 9	Mather’s education ≤ 9	Mather’s education > 9	Very poor/poor	Average	Rich/very rich
	A. Total Effects								
Myopic	0.113 ***	0.123 ***	0.123 ***	0.114 ***	0.113 ***	0.144 ***	0.147 ***	0.112 ***	0.117
	(0.026)	(0.020)	(0.019)	(0.027)	(0.019)	(0.032)	(0.029)	(0.021)	(0.072)
95% CI	[0.061, 0.164]	[0.084, 0.164]	[0.084, 0.161]	[0.060, 0.168]	[0.075, 0.151]	[0.080, 0.208]	[0.089, 0.205]	[0.071, 0.153]	[−0.027, 0.260]
	B. Direct Effects								
Myopic	0.156 ***	0.152 ***	0.159 ***	0.143 ***	0.145 ***	0.194 ***	0.187 ***	0.138 ***	0.250 **
	(0.039)	(0.025)	(0.022)	(0.042)	(0.022)	(0.050)	(0.043)	(0.024)	(0.109)
95% CI	[0.078, 0.234]	[0.103, 0.203]	[0.116, 0.202]	[0.059, 0.226]	[0.101, 0.189]	[0.095, 0.292]	[0.102, 0.272]	[0.090, 0.186]	[0.034, 0.466]
	C. Indirect Effects								
Myopic	−0.047	−0.029	−0.036 *	−0.033	−0.032	−0.056	−0.041	−0.026	−0.142
	(0.036)	(0.025)	(0.021)	(0.042)	(0.020)	(0.050)	(0.042)	(0.022)	(0.096)
95% bootstrap CI	[−0.119, 0.024]	[−0.080, 0.019]	[−0.077, 0.009]	[−0.110, 0.048]	[−0.072, 0.009]	[−0.163, 0.039]	[−0.126, 0.040]	[−0.069, 0.016]	[−0.316, 0.064]
N	8169	10,476	12,007	6568	13,316	5329	3823	13,701	1121

Notes: All regressions include all personal and family characteristics presented in Table 1 and school fixed effects. Standard errors, reported in parentheses, are clustered at the school level. 95% confidence intervals (CIs) are reported in brackets; for indirect effect estimates, 95% bootstrap CIs are reported. *** *p* < 0.01, ** *p* < 0.05, * *p* < 0.1.

## Data Availability

The data supporting reported results are available in the Chinese National Survey Data Archive at: http://www.cnsda.org/index.php?r=prjects/view&id=72810330 accessed on 15 October 2020 and http://www.cnsda.org/index.php?r=projects/view&id=61662993 accessed on 15 October 2020.
